# Acceptance of online therapy for children and adolescents with digital media use disorders: perspectives from child and adolescent psychiatrists and psychotherapists in Germany

**DOI:** 10.1007/s00787-025-02640-w

**Published:** 2025-01-13

**Authors:** K. Busch, G. A. ten Hoor, K. Paschke, R. Thomasius, N. Arnaud

**Affiliations:** 1 German Center for Addiction Research in Childhood and Adolescence (DZSKJ), University Medical Center Hamburg-Eppendorf (UKE), Martinistrasse 52, 20246 Hamburg, Germany; 2 Department of Work and Social Psychology, Faculty of Psychology and Neurosciences, Maastricht University, PO BOX 616, 6200 MD Maastricht, The Netherlands

**Keywords:** Acceptance, Online therapy, Digital media use disorders, Child and adolescent psychiatrists and psychotherapists, Online survey

## Abstract

Online therapies have the potential to improve access to psychological services for individuals in need while alleviating the burden on healthcare systems. However, child and adolescent psychiatrists and psychotherapists (CAPPs) rarely integrate these services into their daily practice. This exploratory study investigates CAPPs’ acceptance of online therapy, with a focus on treating children and adolescents with digital media use disorders (DMUD). The study aimed to examine attitudes toward online therapy for DMUD treatment and to identify barriers and facilitating factors to its implementation. A cross-sectional online survey (5–10 min) was distributed to 1000 members of a German practitioner network, with 142 respondents completing it in full. Attitudes toward online therapy were assessed using adapted versions of the *Attitude towards Telemedicine in Psychiatry and Psychotherapy (ATiPP)* questionnaire. Barriers and facilitating factors were explored using open-ended questions. Descriptive statistics, correlations, and regressions were used to analyze the closed-ended questions, while responses to open-ended questions were categorized. CAPPs reported considerable experience with DMUD, but less experience with online therapy. Attitudes towards online therapy were generally neutral, with less favorable views on its use for DMUD treatment. Key barriers to implementation in outpatient care included technical challenges, lack of personal contact or control, and concerns about data security. Facilitators included access to adequate technical resources, user-friendly and evidence-based programs, interactive tools, and opportunities for regular face-to-face interactions. The results highlight the need to develop online therapy solutions that align practitioners’ needs and acceptance. However, further qualitative and quantitative research with more representative samples is essential for a more comprehensive understanding of this topic.

**Supplementary Information:**

The online version contains supplementary material available at 10.1007/s00787-025-02640-w.

## Introduction

Psychiatric disorders among children and adolescents continue to pose a significant challenge to the healthcare system worldwide [1]. Healthcare providers are facing a pressing need for more staff with expertise in this area [1]. For example, in Germany, a shortage of skilled staff particularly of young professionals seems to strain the healthcare system, with 77% of resident child and adolescent psychiatrists and psychotherapists (CAPPs) aged 50 years or older [2, 3]. Digital mental health interventions (DMHIs) could be used to bridge waiting times and provide and support treatment [4, 5].

DMHIs are digital programs that deliver and support psychiatric and psychotherapeutic treatment via online or mobile platforms with different levels of guidance by practitioners [6]. They aim to recognize, monitor, treat, or alleviate diseases or injuries [7]. Despite the promising role of DMHIs in healthcare, many clinical practitioners appear to be skeptical of DMHIs and hesitant to use them in their daily practice [8]. For example, in a large cross-sectional study (N = 1308) among adult outpatient-care general practitioners, psychotherapists, and physicians in Germany, none of the respondents had ever prescribed DMHIs, and less than one-third planned to prescribe them [9]. Several barriers such as lack of information, patient privacy or safety, and technological or legal uncertainties were identified by clinical practitioners [9–11]. Furthermore, DMHIs are mainly available and approved for adult patients [12]. Even though they show good potential to improve current health care for adolescents [5, 13–15] DMHIs for the youth population are currently largely unavailable.

Meanwhile, more children and adolescents are at risk for problematic digital media use further challenging the healthcare system. This is underlined by the inclusion of the new diagnosis of gaming disorder in the ICD-11 in January 2022 [16]. Digital media use disorders (DMUD) serve as an umbrella term for risky or pathological gaming, social media use, or video streaming [17]. According to a representative survey among 10 to 17-year-olds in Germany, around 6% of children and adolescents fulfill the ICD-11 criteria for pathological gaming or social media use, and another 12–16% report hazardous digital media use [18]. Since gaming disorder is a relatively new clinical diagnosis and problematic use of social media and streaming is still under research, interventions for adolescents are in development, most prominently treating gaming disorders [19]. In this realm, DMHIs may be particularly attractive for the affected patients given their affinity with digital media [20].

The acceptance of DMHIs among healthcare professionals is essential to foster implementation into clinical routines since they can act as gatekeepers to access DMHIs [11]. Acceptance pertains to healthcare providers’ preferences and expectations when considering the provision of DMHIs [21]. Positive attitudes toward DMHIs among healthcare providers have been shown to enhance patient trust and thereby influence their use [22]. Previous studies focused on practitioners’ attitudes and acceptance toward DMHIs in adult care. The Unified Theory of Acceptance and Use of Technology (UTAUT) has been used to study the acceptance of DMHIs. This theory proposes four determinants of user acceptance and behavior, i.e. performance expectancy, effort expectancy, social influence, and facilitating conditions [23]. One study found that in-patient care practitioners in Germany have little experience and indifferent attitudes regarding DMHIs for adults while other studies identified a moderate general acceptance of DMHIs among psychotherapists in training [24, 25].

A large European project focused on issues regarding implementing E-mental health in six European countries [26]. Next to country-specific aspects, all six reported common barriers including lack of knowledge, awareness, or acceptance, concerns about data protection and privacy, and hindering reimbursement structure. Similarly, previous (systematic) reviews identified common aspects reported by practitioners concerning E-mental health interventions such as more training and information to reduce concerns [8, 11, 27], financial reimbursement [8, 11, 28], blended approaches with face-to-face contact/therapeutic relationship [11, 27, 28], additional resources (i.e. new methods, facilities, staff, time) [11, 28], and data security/privacy issues [27, 28]. Mohr et al. [29] argue that DMHIs should be designed and fit into daily practice indicating that all stakeholders (patients and practitioners) closely involved in the implementation of DMHIs for children need to be addressed for increasing acceptance and reducing barriers.

However, research focusing on child and adolescent psychiatry and the specific need of this population is sparse. Results from adult care cannot be simply translated to children and adolescents. For example, working with children and adolescents generally requires a family-centered approach and active caregiver involvement [30]. Caregiver’s consent is mandatory for many medical decisions and the level of guidance likely differs between adolescent and adult patients. In addition, parental views and attitudes towards mental health services can influence treatment uptake among adolescents such that parents act as gatekeepers to treatment access [31]. Further, adolescents typically show less problem awareness, low motivation to change addictive behavior, and poor mental health literacy [32, 33], which can limit seeking professional help. In summary, to improve the care problem, it is essential to provide CAPPs with feasible and high-quality DMHIs specific to the needs of their patient population and that regard important barriers as well as facilitating factors to the implementation to increase CAPPs acceptance.

Therefore, the aim of this study is twofold: Firstly, to explore personal and cognitive factors that are associated with the acceptance of DMHIs for children and adolescents with DMUD among CAPPs in Germany. Secondly, to identify possible barriers and facilitating factors for the integration of DMHIs into daily practice.

## Methods

To meet our study aims, a cross-sectional study in the form of a short online survey (< 10 min) was conducted in which both closed questions were asked to measure attitudes, and personal and cognitive factors, and open-ended questions were asked to identify barriers and facilitating factors.

### Participants and procedures

An online survey was distributed among 1,000 members of the national professional association of medical doctors specialized in child and adolescent psychiatry, psychosomatics, and psychotherapy in Germany (Berufsverband für Kinder- und Jugendpsychiatrie, Psychosomatik und Psychotherapie in Deutschland e.V./BKJPP). The study was deployed using the online survey tool REDCap and included a total of 31 items. The study was advertised through emails sent to the BKJPP members with monthly reminders between March and June 2023. Participation was voluntary and no compensation was offered. Ethics approval was obtained by the local committee at the University Clinic Hamburg-Eppendorf, in accordance with the Declaration of Helsinki. A total of N = 181 licensed CAPPs in Germany provided informed consent and completed the online survey. After excluding 39 respondents with incomplete data, the final sample consisted of 142 participants.

### Measures

#### Demographics

Participants were asked to self-report their age, gender, occupation, and work experience in years as well as their self-rated IT skills/knowledge (1 = very bad to 5 = very good).

#### Self-efficacy, experience, and interest in online therapy and DMUD

Eight items were constructed, two items asking about their feeling to be capable [perceived self-efficacy] to use online therapy and DMUD (“I have a clear understanding of how to use online therapies as a therapeutic tool/DMUD”), four items about experience with online therapy and DMUD (e.g. “I have previously treated children and adolescents with online therapy/DMUD”) and two items about interest in online therapy and DMUD (“I would like to learn more about the use and possibilities of online therapy for children and adolescents/about DMUD”). The reliability of the two items measuring experience with online therapy was excellent (Cronbach’s alpha of 0.95) and for the two items measuring experience with DMUD was sufficient (Cronbach’s alpha of 0.70). One additional item was used to measure prior experience with treatment of addiction among children and adolescents (“I have previous experience of treating children and adolescents with an addiction”). All items were rated on a 5-point Likert scale (1 = strongly disagree to 5 = strongly agree).

#### Attitudes toward using online therapy in general and specifically for DMUD

The Attitude toward Telemedicine in Psychiatry and Psychotherapy (ATiPP) was used to assess participants’ feelings to respond favorably or unfavorable toward the use of online therapy in psychiatry and psychotherapy in general and specifically for treatment of DMUD [34]. For both versions, we used the adapted version by Sander et al. [25] that utilizes the narrowed-down concept of the use of “online therapy” instead of “telemedicine”. In our survey, we used the term “online therapy” to refer to all internet-based or e-mental health applications including apps and programs that you can use on your computer, tablet, or smartphone as well as digital communication tools designed for the treatment of mental illness [25]. The instrument includes eight self-report items with a 5-point Likert scale (1 = strongly disagree to 5 = strongly agree). A higher score indicates a more positive attitude towards the use of online therapy. The original paper reported good reliability (Cronbach’s alpha of 0.83) which was similar to the adapted version (Cronbach’s alpha of 0.73) [25, 34]. In this study, the Cronbach’s alpha was 0.89.

Additionally, to investigate the attitudes towards the use of online therapy specifically for DMUD, we adapted four items of the ATiPP (i.e. items 1, 3, 6, 7) to refer specifically to the treatment of DMUD among children and adolescents. The Cronbach’s alpha of the four items was 0.92. However, the average item correlation was high (0.69–0.83), indicating that the items may overlap. Additionally, the correlation between the adapted ATiPP and the ATiPP adapted for DMUD was high (*r* = 0.81, *p* < 0.001). See Supplementary material 1 for all inter-correlations between the ATiPP items.

#### Barriers and facilitating factors for the implementation of online therapy in psychiatric outpatient care

Four open-ended questions were used to assess possible barriers and facilitating factors for implementing online therapy in an outpatient CAPP setting. The items asked about (1) prerequisites for successful implementation, (2) helpful features of online therapy programs for children and adolescents, (3) perceived barriers to the successful implementation of online therapy in daily practice, and (4) how to overcome these barriers. The questions were inspired by the UTAUT model predictors [23] and were adapted from a previous study [25]. Only four open items were used to minimize the effort for the respondents.

## Data analysis

The data were cleaned to filter out incomplete responses such that only full completers were included. This was done because we obtained datasets that were either fully completed or had at least one of the required instruments missing, which rendered them incomparable to the completed responses and did not allow for multiple imputation methods. Quantitative descriptive analyses of the scale scores (mean values, standard deviations, frequencies, and percentages) as well as correlational analyses and multiple regression analyses were performed using IBM SPSS Statistics, Version 29.0. All assumptions for the regression analyses were tested and met, including skewness, kurtosis, and collinearity. The data exhibited a slight skewness which was not excessive (below 1.0) [35] and we did not correct for multiple testing in line with Bender and Lange [36]. To analyze the four open-ended questions that contained four themes (namely: prerequisites, helpful features, barriers, and solutions to overcome the barriers) used in this survey, we created categories and codes based on the data we had collected. We then sorted the phrases into each category [37]. The respondents were counted based on their response to an open-ended question and one answer could be allocated to one or more categories. Raw data from the short text responses were initially organized and analyzed by the first author (KB). Both GAtH and NA independently checked the codes for data triangulation and conflicting results were discussed. Analyses were conducted using MAXQDA [38] and Microsoft Excel [39].

## Results

Participants’ characteristics are depicted in Table 1. The majority of respondents were CAPPs (93%) with a mean age of 53.5 years and an average of 22.3 years of professional experience. Both age (*r* = − 0.23, *p* = 0.006) and professional experience (*r* = − 0.24, *p* = 0.004) were significantly negatively correlated with IT skills/knowledge. In addition, age and professional experience were highly positively correlated (*r* = 0.85, *p* < 0.001). Half of the respondents rated their IT skills/knowledge as neutral (50%), whereas around 37% indicated it was good to very good, and around 13% stated it was bad to very bad.

**Table 1:** Participant characteristics

	*N*	%
Gender		
Male	47	33.1
Female	94	66.2
Diverse	1	0.7
Occupation		
Medical doctor for child and adolescent psychiatry and psychotherapy	132	93
Training assistant in child and adolescent psychiatry and psychotherapy	3	2.1
Licensed psychotherapist for children and adolescents	3	2.1
Other medical doctor	1	0.7
In training to become child and adolescent psychotherapist	1	0.7
Psychologist	1	0.7
Pedagogue	1	0.7
Self-reported IT skills and knowledge		
Very bad	1	0.7
Bad	17	12
Neutral	71	50
Good	50	35.2
Very good	3	2.1
	M (range)	SD
Age (in years)	53.5 (27–69)	8.4
Professional experience (in years)	22.3 (2–40)	8.5

*N* = 142

### Self-efficacy, experience, and interest in online therapy and DMUD

Around half of the respondents (51%) reported to “have a clear understanding of how to use online therapies as a therapeutic tool”, whereas 16% were unsure and 34% did not agree to have a clear understanding. Similarly, less than half (41%) of the CAPPs have had experience using online therapy in therapy among children and adolescents. In general, around two-thirds (67%) were interested “to learn more about the use and possibilities of online therapy for children and adolescents”, however, 16% of CAPPs were unsure.

Concerning DMUD, almost all of the respondents indicated to have a “clear understanding of DMUD among children and adolescents” (97%). Around 87% have had prior experience in treating children and adolescents with DMUD. Importantly, the CAPPs in this sample were experienced in the treatment of addiction, with 85% indicating to have treated children and adolescents with addiction before. In general, the interest to “learn more about DMUD in children and adolescents” was high (85% partially or fully agreed). See Supplementary material 2 for full item results.

### Attitudes towards online therapy for children and adolescents in general and with DMUD

The mean scale scores of both the ATiPP and the adapted version concerning DMUD tended towards the middle of the scale (M_ATiPP_ = 3.56, SD = 0.81; M_ATiPP_DMUD_ = 3.10, SD = 1.00) which ranged from 1 to 5, with 1 = totally disagree and 5 = strongly agree to statements about usefulness of online therapy for adolescents. Both ATiPP scores did not correlate with age, professional experience, or IT skills. At the item level, around half (48%) (strongly) agreed that ‘an effective treatment of patients with mental illness via online therapy is possible’, whereas only 34% agreed with the same statement when adapted for use in the treatment of DMUD. Similarly, around two-thirds of the participants agreed that ‘generally, online therapy is a good addition to medical services’ (66%). Contrarily, less than half (44%) agreed when the same item was adapted for use in the treatment of DMUD and 24% of respondents were unsure. In general, around 20 to 30% of participants replied with “neutral” to all items, indicating uncertainty with regard to the evaluation of using online therapy in the specific context. For full ATiPP and ATiPP_DMUD_ results on the item level, see Supplementary material 3.

### Bivariate correlation analyses of all study variables

Bivariate correlations revealed statistically significant correlations between a more positive attitude toward online therapy and higher self-efficacy with using online therapy, lower self-efficacy with DMUD, more experience with using online therapy, and a higher interest in learning more about online therapy. Further, the attitude towards using online therapy for the treatment of DMUD, self-efficacy with using online therapy, self-efficacy with DMUD, and interest in learning more about online therapy and DMUD were significantly correlated. For correlations among all study variables see Supplementary material 4.

### Associations of attitudes and personal and cognitive factors

A stepwise multiple linear regression analysis was conducted to examine the associations between attitudes toward using online therapy (ATiPP mean score) and personal and cognitive factors. Cognitive factors included self-efficacy and interest in both online therapy and DMUD whereas personal factors included age, work experience, IT skills, and experience with online therapy and treatment of DMUD and addictive disorders in general. The analysis revealed statistically significant positive relationships between the attitudes towards using online therapy and both the interest to learn more about online therapy and self-efficacy with using online therapy (see Table 2). The final model including only interest and self-efficacy with online therapy explained 48% of the observed variance of the attitude toward using online therapy among the CAPPs in this sample.

**Table 2:** Multiple regression analyses of the attitudes toward using online therapy for children and adolescents (with DMUD)

Variable	*B*	*SE B*	*β*	*t*	*p*
Attitude OT					
(Constant)	1.59	0.20		8.13	< 0.001
Self-efficacy OT	0.12	0.04	0.16	2.68	**0.008**
Interest in OT	0.43	0.04	0.66	10.67	** < 0.001**
Corrected R^2^	**0.48**				
Attitude OT for DMUD					
(Constant)	0.96	0.05		3.63	< 0.001
Self-efficacy OT	0.12	0.06	0.13	1.99	**0.049**
Interest in OT	0.47	0.05	0.59	8.71	** < 0.001**
Corrected R^2^	**0.38**				

Bold values indicate significant results of main variables of interest, only significant predictors are depicted

*β* standardized beta, *B* unstandardized beta, *SE B* standard error of unstandardized beta, *t* t-test statistic, *DMUD* digital media use disorders, *OT* online therapy

The same model was calculated specifically for DMUD (ATiPP_DMUD_ mean score). Similar to the first model, attitude concerning using online therapy for DMUD was statistically significantly related to both the interest to learn more about online therapy as well as self-efficacy with using online therapy (see Table 2). The final model including only interest and self-efficacy with online therapy explained 38% of the observed variance of the attitude toward using online therapy for treatment of DMUD among the CAPPs in this sample.

### Potential facilitating factors and barriers to the implementation of online therapy for children and adolescents

The answers to the different themes overlapped, for example, some respondents mentioned data security as a prerequisite whereas others mentioned it as a potential barrier to the implementation of online therapy. Where we aimed to concisely report our findings here, more detailed tables on the overlap of the themes and categories can be found in Supplementary material 5.

### Facilitating factors and helpful features of online therapy in outpatient care

Four categories with potential facilitating factors were identified by the researchers as well as aspects concerning the design and features of the online therapy. Regarding the *therapeutic aspects* (i.e. contact with patients, the inclusion of parents), 32% (N = 25) opted for online therapy with regular face-to-face contact, and 5% (N = 4) recommended working closely with parents or legal guardians in the process. For example, one respondent mentioned online therapy “Only as an addition to ongoing face-to-face therapy” and another stated that “Online therapy could also be very useful for caregivers”. Further, a strong therapeutic relationship was highlighted by the CAPPs for the implementation of online therapy for patients (4%, N = 3) as well as interactive tools to communicate with the patients (3%, N = 2). Secondly, *technical aspects* (i.e. technical equipment, Wi-Fi connection) such as good technical equipment (17%, N = 13), a stable internet connection (6%, N = 5), and training about online therapy and specifically its technical application (8%, N = 6) was another important aspect for the CAPPs. Regarding *legal or regulatory* aspects (i.e. security and validity of programs), data protection (9%, N = 7), availability of evidence-based and validated programs (9%, N = 7) and basis for billing (4%, N = 3) were the most common. For example, one respondent highlighted the “Comprehensive validation of the programs”. Lastly, *organizational aspects* (i.e. setting, resources, training) such as an undisturbed space for the use of online therapy programs (4%, N = 3) or acceptance among staff and patients (3%, N = 2) were highlighted. For example, one respondent said, “That the kids have a disturbance-free space in which the therapy can take place”. Interestingly, one person mentioned the advantage for patients who live far from the practice and another mentioned sufficient assistance.

According to the CAPPs, the design of online therapy should be user-friendly and have high usability (20%, N = 12) as well as an appealing design (14%, N = 8). Contents and features mentioned were among others psycho-educational content (5%, N = 3) followed by a clear structure (e.g. modules) within the online therapy intervention (5%, N = 3) and diaries as visualization of the progress (5%, N = 3). Interactive features such as video calls or chat (19%, N = 11) as well as feedback and insight into the online therapy use of patients were highlighted by 14% (N = 8). For example, “Opportunities to interact with the practitioner can be set (by the practitioner)” was mentioned. Interestingly, one person mentioned a virtual waiting room to discuss things next to therapy and another mentioned the importance of using online therapy only for stable and familiar patients. See Tables 3 and 4 for a summary.

**Table 3:** Prerequisites and facilitating factors for implementation of online therapy

Categories	Codes	*N*	%
*Therapeutic aspects*			
	Regular face-to-face contact	25	32
	Specific concepts (e.g. different disorders, structure of program)	4	5
	Integration of parents/legal guardians	4	5
	Age-appropriate online therapy programs	3	4
	Strong therapeutic relationship and clear goals	3	4
	Interactive tools for contact between patient and therapist	2	3
	Handling emergencies (e.g. acute suicidality)	2	3
*Technical aspects*			
	Good technical equipment	13	17
	Training of CAPPs, specifically technical aspects	6	8
	Stable internet connection/stable network	5	6
	Easy handling, user-friendly design	5	6
	Sufficient knowledge of technical aspects	3	4
	Easy access, e.g. via smartphone	3	4
	IT support/hotline	1	1
*Legal/regulatory aspects*			
	Data protection	7	9
	Evidence-based, validated programs	7	9
	Regulatory aspects	4	5
	Basis for billing	3	4
	Secure platforms to administer online therapies	2	3
	Duty of confidentiality/parental consent	1	1
*Organizational aspects*			
	Undisturbed space in facilities/for patients at home	3	4
	Acceptance by staff and patients	2	3
	Better controllable in an in-patient setting	2	3
Participants with answer		77	54
Participants without answer		65	46
Total number of participants		142	100

**Table 4:** Features and perceived benefits for implementation of online therapy

Categories	Codes	*N*	%
*Design*			
	User-friendly, good usability	12	20
	Attractive design	8	14
*Therapeutic aspects*			
	Interactive functions (e.g. video, chat, group chat)	11	19
	Insight into app progress/feedback	8	14
	Add-on to face-to-face therapy	4	7
	Undisturbed space in facilities/for patients at home	2	3
	Usage/time tracking	1	2
	Age-appropriate online therapies	1	2
	Handling emergencies (e.g. acute suicidality)	1	2
*Technical aspects*			
	Stable internet connection/stable network	5	8
	Good technical equipment	2	3
	IT support/hotline	1	2
*Legal/regulatory aspects*			
	Data protection	4	7
	Digital informed consent	1	2
	Evidence-based, validated programs	1	2
*Content-related aspects*			
	Psychoeducational content	3	5
	Clear structure/modules	3	5
	Diaries/visualization of progress	3	5
	Relaxation exercises	1	2
	Questionnaires	1	2
	Skills catalog	1	2
Participants with answer		59	42
Participants without answer		83	58
Total number of participants		142	100

### Barriers and solutions to overcome in the implementation of online therapy in outpatient care

Concerning barriers, four categories could be identified from the CAPPs responses. Firstly, several *therapeutic aspects* were identified including the lack of therapeutic relationships in online therapies (12%, N = 9), little commitment of patients (9%, N = 7), and lack of feedback or control over the use of the online therapy (8%, N = 6). Concerning digital media use in therapy, 9% (N = 7) of CAPPs had concerns about media use for therapeutic purposes, because they were either against the use of digital media by young people in general or specifically for patients with DMUD. For example, one respondent critically stated that online therapy is “Even more media when it is supposed to bring away”. Secondly, *technical aspects* including technical problems (12%, N = 9), unstable internet connection in the facilities (12%, N = 9), and lack of technical equipment, also among patients (10%, N = 8) were reported. *Legal or regulatory aspects* included data protection concerns (10%, N = 8), lack of billability (4%, N = 3) as well as the rigidity of the healthcare system (5%, N = 4). For example, one respondent stated “Legal texts—it is not provided for, so it does not exist”. Lastly, several CAPPs stated *organizational aspects* concerning resources such as lack of time/effort (8%, N = 6), lack of knowledge about online therapy (3%, N = 2) as well as little willingness to change the established practice due to reservations about digital media (3%, N = 2) or (additional) financing (3%, N = 2). In terms of solutions on how to overcome the barriers, CAPPs stated training requirements (15%, N = 10), improved data protection such as secure platforms to work with (12%, N = 8) as well as a standardized catalog of all available digital interventions to find specific programs for children and adolescents when needed (11%, N = 7). For example, one respondent mentioned that “Even more information, especially for practitioners” is needed. See Tables 5 and 6 for a summary.

**Table 5:** Barriers to implementation of online therapy

Categories	Codes	*N*	%
*Therapeutic factors*			
	Lack of therapeutic relationship/little personal	9	12
	Little commitment from patients	7	9
	Against more digital media for adolescents/against digital media for treatment of DMUD	7	9
	Lack of control or feedback about online therapy use	6	8
	Low compliance of patients	4	5
	Handling emergencies (e.g. acute suicidality)	3	4
	Lack of non-verbal communication	3	4
	Low acceptance of CAPPs/patients	2	3
	Add-on to face-to-face therapy	1	2
	Age-appropriate online therapies	1	2
*Technical equipment*			
	Unstable internet connection	9	12
	Technical problems	9	12
	Lack of technical equipment	8	10
	IT support/hotline	1	1
*Legal/regulatory aspects*			
	Data protection	8	10
	Rigid healthcare system	4	5
	Billability	3	4
	Inclusion of parental/legal guardian	2	3
	Evidence-based, validated programs	1	2
	Digital informed consent	1	2
*Organizational aspects*			
	Lack of time/effort	6	8
	Lack of knowledge about online therapies	2	3
	Little willingness to change around CAPPs	2	3
	Financing (e.g. personnel)	2	3
	Training of CAPPs	1	2
Participants with answer		78	55
Participants without answer		64	45
Total number of participants		142	100

**Table 6:** Factors to overcome barriers to implementation of online therapy

Categories	Codes	*N*	%
*Organizational aspects*			
	Training of CAPPs	10	15
	Standardized catalog of available digital interventions	7	11
	Reduction of reservations regarding online therapy	4	6
	Time/effort for online therapy	2	3
	Access to test digital interventions themselves	2	3
	Costs	1	2
*Legal/regulatory aspects*			
	Data protection	9	14
	Evidence-based, validated programs	6	9
	Billability/compensation	4	6
	Duty of confidentiality/parental consent	1	2
*Technical aspects*			
	Stable internet connection	6	9
	Sufficient technical equipment	3	5
	IT support/hotline	1	2
	Sufficient knowledge of technical aspects	1	2
	Easy handling, user-friendly	1	2
*Therapeutic factors*			
	Personal contact with patients	3	5
	Therapeutic support for online therapies	1	2
Participants with answer		66	46
Participants without answer		76	54
Total number of participants		142	100

## Discussion

The purpose of this exploratory study was to gain a better understanding of the acceptance of online therapy in general and specifically for the treatment of DMUD. Almost all of the CAPPs in this sample reported considerable experience and sufficient self-efficacy with the treatment of DMUD among children and adolescents. In comparison, respondents reported less experience and lower self-efficacy with using online therapy in the therapeutic setting. Attitudes toward using digital tools for therapeutic purposes, in general, were mostly indifferent and slightly less favorable for specific treatment of DMUD. Several barriers and facilitating factors for implementing online therapy targeting therapeutic, technical, legal/regulatory, and organizational aspects were identified.

In line with previous research on the acceptance of e-health in adult care [25, 40, 41], this study identified a generally moderate degree of acceptance towards DMHIs for children and adolescents. However, previous studies identified higher acceptance for specific types of DMHIs [24, 42] and some degree of heterogeneity among care providers. For example, cognitive-behavioral therapists show less skepticism compared to psychodynamic-oriented therapists and DMHIs often utilize strategies with a cognitive-behavioral therapy basis potentially leading to differing preferences [43, 44]. These studies indicate that concerning DMHIs, there is a need to study various aspects of effectiveness, not limited to treatment efficacy as typically tested in clinical trials. In this context, investigating implementation-related factors is important because it can support the long-term use and effectiveness of DMHIs by revealing specific aspects for successful implementation in the care setting. As highlighted in the ImpleMentAll Study [21], acceptance and engagement are two central aspects of effectiveness in a sense beyond treatment efficacy.

We found that CAPPs’ attitudes were associated with the interest to learn more about online therapy options and perceived self-efficacy. Thus, to increase acceptance of online therapy, it could be helpful to raise advertisements and provide more training options for CAPPs to enhance self-efficacy with new digital tools and make DMHIs more attractive for use in daily practice [45]. However, it is important to note that the aforementioned association is weak in predicting potential actions, especially as our exploratory analysis considered various personal and cognitive variables related to attitudes. Nevertheless, examining the attitudes is an important basis for not only explaining current acceptance of online therapy but also for developing targeted actions that can impact acceptance.

Several barriers to implementation in adolescent outpatient care were identified. Lack of personal contact throughout implementing DMHIs to strengthen the therapeutic relationship was highlighted by the respondents aligning with previous findings [11, 24, 27, 28]. Interestingly, a narrative review looked at the therapeutic alliance in DMHIs specifically and found that the association between digital therapeutic alliance and therapeutic outcomes is less strong. However, it might play an essential role in the adherence and engagement of users using DMHIs [46]. Similarly, Khanna and Carper [47] pointed out that future studies need to investigate what level and quality of involvement by practitioners is needed for DMHIs for adolescents, in this case, with anxiety. Further, similar to previous studies, technical aspects included unstable internet connections and technical equipment [11, 28]. More resources for outpatient CAPPs to fully integrate technical systems with already pre-existing ones could be helpful [28]. In addition, age-appropriate online therapy programs and how to act in emergencies are particularly important in the context of child and adolescent psychiatry and psychotherapy. Interactive tools or elements to communicate with the patients and an emergency kit with step-by-step instructions on what to do could overcome this barrier [48].

As pointed out by several respondents, using digital media to treat DMUD may seem counterintuitive given their problematic media use in the first place. However, the approach to making contact and motivating adolescents for therapy via digital tools like app-based formats shows potential [20] and may even be received particularly positively by DMUD patients. There are however also several counterarguments to incorporating digital media such as smartphones into therapy. For example, the fear of misuse and lack of control were two aspects mentioned by CAPPs in this survey. The results of this survey identified some concerns, however, the majority of CAPPs did not highlight this issue specifically. Future research is needed to shed light on the potential benefits as well as drawbacks of online therapy with this particular target group. One current example is the ongoing Res@t study. This study investigates the use of app-based training next to treatment as usual targeting problematic gaming, social media, and video-streaming use among adolescents [48].

Frequently mentioned facilitating factors for the implementation of online therapy in outpatient care for adolescents included such factors as regular face-to-face contact with the patients. This is in line with previous research indicating that human support seems to enhance the effectiveness and acceptance of DMHIs [45, 49, 50], specifically for patients with greater symptom severity [51]. Further, good technical equipment and training are necessary prerequisites for implementation, which was highlighted by previous research [9, 52, 53]. Interestingly, the respondents emphasized the role and level of involvement of caregivers as an important aspect. This issue is specific to child and adolescent psychiatry; however, it was surprisingly not an aspect mentioned by the majority. This might be due to the open-ended format applied. Nevertheless, the parental role should be clearly defined when developing DMHIs for children and adolescents; a strategy may be to target both parents and their children in online therapy programs such as the app-based Res@t program [54] to support therapeutic processes. Interestingly, a recent review highlighted the lack of platforms for collaborating in digital programs, for example as a family [55]. A lack of individual privacy and safety when working together with multiple users in such programs was identified.

Our findings have clear practical implications for outpatient child and adolescent psychiatric care. The CAPPs in our study indicated that in addition to the barriers and facilitating factors identified in previous research, receiving feedback and having insight into online therapy programs, as well as having specialized programs tailored to different age groups would be beneficial. Including features to track and view the progress in DMHIs could support the acceptance among CAPPs and may give them a sense of control when integrating them into their clinical work. Future studies should further investigate the impact of feedback on practitioners’ acceptance and develop interventions tailored to different age groups. The role of parents as gatekeepers to their children's access to care was mentioned by some respondents. The findings indicate that involving caregivers in their children’s therapy by, for example offering DMHIs for them next to the patients themselves and exploring their preferences might improve their acceptance. For example, previous research indicates that parents may prefer face-to-face over blended approaches, but still, they accept them more when they perceive them as effective [56]. Future research should focus on the different stakeholders’ needs and preferences to enhance the care for adolescents. One way to overcome the lack of self-efficacy and experience with using online therapy could be to implement an official catalog with all available digital health applications. While such a directory has already been established (in German: Digitale Gesundheitsanwendungen, DiGA), the availability of specific applications for the youth population is currently underdeveloped [57]. By improving current evidence of the effectiveness of DMHIs, and focusing on the usability and data security of DMHIs, the acceptance among CAPPs could be enhanced [58]. Especially given the reservations and skepticism identified in this study, future studies should investigate how to motivate and educate CAPPs to use and implement DMHIs. The active involvement in the development of such DMHIs might support acceptance and ease its implementation among CAPPs as [59] has previously shown.

## Limitations and future considerations

Research on effective treatment including implementation studies is largely lacking for the youth population. The results from this study can inform about possible pointers for future research to improve mental health care. However, the following study limitations should be considered. Firstly, convenience sampling was conducted via a mailing pool of CAPPs in Germany (BKJPP) with a 14.2% respondent rate (N = 142/1000) which is not representative of all CAPPs in Germany. However, according to the German Medical Association [3], 1063 outpatient CAPPs were employed in 2022 with a median age of around 55 years, which is similar to the mean age in this sample. Additionally, the collection of limited demographic data constrained the ability to thoroughly characterize the respondents in this sample. Further, some respondents were either strongly against or strongly in favor of online therapy and this might have led them to replying to the survey leading to potential distortion. Future research should investigate a representative sample and control for such effects. Secondly, the study did not differentiate between different types of DMHIs but rather used a broader definition of online therapy. This was done to assess the overall acceptance in an exploratory way. However, the acceptance might differ depending on, e.g. guided- vs. stand-alone interventions. Additionally, an updated taxonomy of types of DMHIs was proposed to group DMHIs [60]. Future research distinguishing between the different types and different care providers (i.e. medical doctors, psychological psychotherapists) should be conducted. Thirdly, only half of the participants reported practical experience with online therapy such that the results partially remain rather hypothetical. We did not provide all participants with an example of online therapy interventions beforehand, which could have established a more uniform knowledge base. This could be a confounder as prior research has shown that sufficient knowledge and digital literacy are necessary for the successful implementation of DMHIs [53]. Lastly, the responses to the open items were very precise and short with limited scope for interpretation. Even though some responses were very clear and understandable such as therapeutic or technical aspects, other categories, particularly legal or regulatory aspects were rather unclear as to what specific hurdles they entail for future implementation of online therapy due to lack of details in the responses. Future research should also examine some of the categories identified in this study in more detail. For example, it remained unclear what legal and regulatory aspects specifically hinder implementation and why CAPPs think so.

## Conclusions

Exploring the acceptance and reservations about online therapy is crucial to inform the future implementation of DiGA and other online therapy formats. CAPPs can act as gatekeepers for access to online therapy and play a pivotal role in the successful implementation of outpatient care. As such, their attitudes and needs are essential when developing online therapy for children and adolescents. This study identified rather indifferent attitudes towards using online therapy in general and specifically for treatment of DMUD among CAPPs. In addition, many barriers and facilitating factors were identified that go beyond the individual level of the CAPPs themselves and should be respected when implementing online therapy into child and adolescent psychiatry. DMHIs for children and adolescents should be easy to use, secure and billable, easy to implement technically, and interactive. It appears that outpatient CAPPs value regular face-to-face contact throughout using DMHIs with adolescents such that they can decide on an individual basis and feel connected to their patients. Future research could investigate the needs of other stakeholders such as the patients or caregivers concerning personal contact. Furthermore, while this study examined acceptance among CAPPs using a cross-sectional survey, additional qualitative studies and studies with representative samples are suggested to better understand and address the different barriers and prerequisites to implementation in outpatient care specifically.

## Supplementary Information

Below is the link to the electronic supplementary material.

Supplementary file1 (PDF 719 KB)

## Data Availability

The data that support the findings of this study are available from the corresponding author upon reasonable request.
