# Supplementary material for: Acceptance of online therapy for children and adolescents with digital media use disorders: perspectives from child and adolescent psychiatrists and psychotherapists in Germany
Source: Eur Child Adolesc Psychiatry. 2025 Jan 13;34(7):2215–27. doi: 10.1007/s00787-025-02640-w (PMC12334523; doi:10.1007/s00787-025-02640-w)
Supplement: Supplementary file 1 — Supplementary file1 (PDF 719 KB) [file 787_2025_2640_MOESM1_ESM.pdf]

Acceptance of Online Therapy for Children and Adolescents with Digital Media Use Disorders: Perspectives from Child and Adolescent Psychiatrists and Psychotherapists in Germany

European Child and Adolescent Psychiatry

Katharina Busch[1], Gill A. ten Hoor[2], Kerstin Paschke[1], Rainer Thomasius[1], & Nicolas Arnaud[1]

[1] German Center for Addiction Research in Childhood and Adolescence (DZSKJ), University Medical Center Hamburg-Eppendorf (UKE), Martinistrasse 52, D-20246 Hamburg, Germany

[2] Maastricht University, Department of Work & Social Psychology, Faculty of Psychology and Neurosciences, PO BOX 616, 6200MD, Maastricht, The Netherlands

Correspondence to Katharina Busch (ka.busch@uke.de)

Supplementary Material 1: Inter-item correlations between both the ATiPP and the adapted ATiPP items

Supplementary Material 2: Frequency of responses to items on self-efficacy, experience, and interest in online therapy and digital media use disorders

Supplementary Material 3: Child and adolescent psychiatrists and psychotherapists' attitude on outpatient care using online therapy

Supplementary Material 4: Descriptive statistics and correlations of study variables for online therapy and digital media use disorders

Supplementary Material 5: Overlap of themes and categories in the open-ended questions and answers

Supplementary Material 1: Inter-item correlations between both the ATiPP and the adapted ATiPP items

Supplementary Table 1a: Inter-Correlations between all ATiPP items

Inter-Item-Correlation Matrix

	atipp_1	atipp_2	atipp_3	atipp_4	atipp_5	atipp_6	atipp_7	atipp_8
atipp_1	1.00	.57	.70	.55	.60	.69	.69	.19
atipp_2	.57	1.00	.57	.56	.62	.58	.49	.21
atipp_3	.70	.57	1.00	.59	.64	.66	.60	.13
atipp_4	.55	.56	.59	1.00	.63	.54	.45	.23
atipp_5	.60	.62	.64	.63	1.00	.64	.66	.25
atipp_6	.69	.58	.66	.54	.64	1.00	.73	.12
atipp_7	.69	.49	.60	.45	.66	.73	1.00	.20
atipp_8	.19	.21	.13	.27	.25	.12	.20	1.00

Note. ATiPP = Attitude towards Telemedicine in Psychiatry and Psychotherapy.

Supplementary Table 1b: Inter-Correlations between all adapted ATiPP items

Inter-Item-Correlation Matrix

	atipp_1_DMUD	atipp_2_DMUD	atipp_3_DMUD	atipp_4_DMUD
atipp_1_DMUD	1.00	.83	.77	.75
atipp_2_DMUD	.83	1.00	.71	.69
atipp_3_DMUD	.77	.71	1.00	.73
atipp_4_DMUD	.75	.69	.73	1.00

Note. ATiPP = Attitude towards Telemedicine in Psychiatry and Psychotherapy. *DMUD* = digital media use disorders.

Supplementary Material 2: Frequency of responses to items on self-efficacy, experience, and interest in online therapy and digital media use disorders

Supplementary Table 2a. I have a clear understanding of how to use online therapies as a therapeutic tool.

	N	**%**	**Cumulative %**
(1) Totally disagree	8	5.6	5.6
2	40	28.2	33.8
3	22	15.5	49.3
4	55	38.7	88
(5) Totally agree	17	12	100
Total	142	100	

Note. N= 142. Original item in German: „*Ich habe eine klare Vorstellung davon, wie ich Onlinetherapien als therapeutisches Mittel einsetzen kann.*"

Supplementary Table 2b. I have previous experience with the use of online therapy with patients in the workplace.

	N	**%**	**Cumulative %**
(1) Totally disagree	43	30.3	30.3
2	30	21.1	51.4
3	6	4.2	55.6
4	35	24.6	80.3
(5) Totally agree	28	19.7	100
Total	142	100	

Note. N= 142. Original item in German: „*Ich habe bereits Erfahrungen mit dem Einsatz von Onlinetherapien für Patient:innen im Rahmen meiner Tätigkeit gemacht.*"

Supplementary Table 2c. I have previously treated children and adolescents with online therapy.

	N	**%**	**Cumulative %**
(1) Totally disagree	65	45.8	45.8
2	15	10.6	56.3
3	4	2.8	59.2
4	29	20.4	79.6
(5) Totally agree	29	20.4	100
Total	142	100	

Note. N= 142. Original item in German: „*Ich habe bereits Kinder und Jugendliche mit einer Onlinetherapie behandelt.*"

Supplementary Table 2d. I would like to learn more about the use and possibilities of online therapy for children and adolescents.

	N	**%**	**Cumulative %**
(1) Totally disagree	12	8.5	8.5
2	12	8.5	16.9
3	23	16.2	33.1
4	49	34.5	67.6
(5) Totally agree	46	32.4	100
Total	142	100	

Note. N= 142. Original item in German: „*Ich würde gerne mehr über den Einsatz und die Möglichkeiten von Onlinetherapien für Kinder und Jugendlichen erfahren.“*

Supplementary Table 2e. I have a clear understanding of digital media use disorders among children and adolescents.

	N	**%**	**Cumulative %**
(1) Totally disagree	0	0	0
2	0	0	0
3	5	3.5	3.5
4	66	46.5	50
(5) Totally agree	71	50	100
Total	142	100	

Note. N= 142. Original item in German: „*Ich habe eine klare Vorstellung davon, was medienbezogene Störungen bei Kindern und Jugendlichen sind.“*

Supplementary Table 2f. I have previous experience with digital media use disorders with patients in the workplace.

	N	**%**	**Cumulative %**
(1) Totally disagree	0	0	0
2	2	1.4	1.4
3	2	1.4	2.8
4	55	38.7	41.5
(5) Totally agree	83	58.5	100
Total	142	100	

Note. N= 142. Original item in German: „*Ich habe bereits Erfahrungen mit dem Störungsbild medienbezogene Störungen bei Patient:innen im Rahmen meiner Tätigkeit gemacht.“*

Supplementary Table 2g. I have previously treated children and adolescents with a digital media use disorder.

	N	%	Cumulative %
(1) Totally disagree	3	2.1	2.1
2	10	7.0	9.2
3	11	7.7	16.9
4	53	37.3	54.2
(5) Totally agree	65	45.8	100
Total	142	100	

Note. N= 142. Original item in German: *„Ich habe bereits Kinder und Jugendliche mit einer medienbezogenen Störung behandelt.“*

Supplementary Table 2h. I would like to learn more about digital media use disorders in children and adolescents.

	N	%	Cumulative %
(1) Totally disagree	0	0	0
2	7	4.9	4.9
3	15	10.6	15.5
4	57	40.1	55.6
(5) Totally agree	63	44.4	100
Total	142	100	

Note. N= 142. Original item in German: *„Ich würde gerne mehr über medienbezogene Störungen bei Kindern und Jugendlichen erfahren.“*

Supplementary Table 2i. I have previous experience of treating children and adolescents with an addiction.

	N	%	Cumulative %
(1) Totally disagree	3	2.1	2.1
2	11	7.7	9.9
3	7	4.9	14.8
4	34	23.9	38.7
(5) Totally agree	87	61.3	100
Total	142	100	

Note. N= 142. Original item in German: *„Ich habe bereits Erfahrungen mit der Behandlung von Kindern und Jugendlichen mit einem Abhängigkeitssyndrom gemacht.“*

Supplementary Material 3: Child and adolescent psychiatrists and psychotherapists' attitude on outpatient care using online therapy

Supplementary Table 3a. Attitude towards online therapy (ATiPP)

**Attitude towards online therapy (ATiPP)**	**[1] Totally disagree**	**[2]**	**[3]**	**[4]**	**[5] Totally agree**	**Total**
Generally, online therapy is a good addition to the medical services.	6 (4.2%)	23 (16.2%)	19 (13.4%)	50 (35.2%)	44 (31%)	142 (100%)
For psychiatric or psychotherapeutic issues or mental illness, patient information via online therapy is very helpful.	6 (4.2%)	9 (6.3%)	31 (21.8%)	59 (41.5%)	37 (26.1%)	142 (100%)
An effective treatment of the patients with mental illness via online therapy is possible.	9 (6.3%)	29 (20.4%)	36 (25.4%)	57 (40.1%)	11 (7.7%)	142 (100%)
The bridging of the waiting time for an appointment in psychiatry/ psychotherapy by using online therapy is a sensible option.	6 (4.2%)	12 (8.5%)	28 (19.7%)	57 (40.1%)	39 (27.5%)	142 (100%)
Aftercare and stabilization after a presence therapy by a psychiatrist or psychotherapist through contact via online therapy is realizable.	6 (4.2%)	15 (10.6%)	28 (19.7%)	67 (47.2%)	26 (18.3%)	142 (100%)
I would absolutely recommend my patients with psychiatric or psychotherapeutic treatment needs an online therapy, if such was to be offered for the clinical picture.	14 (9.9%)	25 (17.6%)	48 (33.8%)	41 (28.9%)	14 (9.9%)	142 (100%)
In addition to a face-to-face therapy, an accompanying online therapy is sensible.	6 (4.2%)	12 (8.5%)	31 (21.8%)	66 (46.5%)	27 (19%)	142 (100%)
An online therapy for mental illness can only work effectively with live contact with a therapist through video calling and email or chat.	6 (4.2%)	20 (14.1%)	33 (23.2%)	62 (43.7%)	21 (14.8%)	142 (100%)

Supplementary Table 3b. Attitude towards online therapy (ATiPP) adapted for digital media use disorders (DMUD)

**Attitude towards online therapy (ATiPP) adapted for DMUD**	**[1] Totally disagree**	**[2]**	**[3]**	**[4]**	**[5] Totally agree**	**Total**
Generally, online therapy is a good addition to the medical services for patients affected by DMUD.	14 (9.9%)	31 (21.8%)	34 (23.9%)	52 (36.6%)	11 (7.7%)	142 (100%)
An effective treatment of the patients with DMUD via online therapy is possible.	12 (8.5%)	35 (24.6%)	47 (33.1%)	39 (27.5%)	9 (6.3%)	142 (100%)
I would absolutely recommend my patients with DMUD an online therapy, if such was to be offered for the clinical picture.	21 (14.8%)	30 (21.1%)	44 (31%)	37 (26.1%)	10 (7%)	142 (100%)
In addition to a face-to-face therapy, an accompanying online therapy is sensible for patients with DMUD.	9 (6.3%)	22 (15.5%)	28 (19.7%)	67 (47.2%)	16 (11.3%)	142 (100%)

Supplementary Material 4: Descriptive statistics and correlations of study variables for online therapy and digital media use disorders

Supplementary Table 4a Descriptive statistics and correlations of study variables for online therapy

Means, Standard Deviations, and Correlations of Demographics, Attitudes and Online Therapy items

	M	SD	Correlations							
			1.	2.	3.	4.	5.	6.	7.	8.
1. Age	53.5	8.45	-							
2. Experience	22.3	8.48	0.85**	-						
3. IT knowledge/ skills	2.26	0.72	-0.23**	-0.24**	-					
4. Self-efficacy with OT	3.23	1.15	0.01	0.01	0.19*	-				
5. Experience with OT	2.71	1.58	-0.11	-0.07	0.16	0.66**	-			
6. Interest in OT	3.74	1.24	-0.27**	-0.16	0.09	0.15	0.18*	-		
7. Attitude OT	3.56	0.81	-0.15	-0.13	0.03	0.26**	0.28**	0.68**	-	
8. Attitude OT for DMUD	3.10	1.00	-0.15	-0.13	0.06	0.22**	0.14	0.61**	0.81**	-

Note. *N*= 142. **p* < .05. ***p* <. 01. *OT* = Online Therapy. *DMUD* = Digital media use disorder.

Supplementary Table 4b. Descriptive statistics and correlations of study variables for digital media use disorders

Means, Standard Deviations, and Correlations of Demographics, Attitudes and DMUD items

	M	SD	Correlations 1.	2.	3.	4.	5.	6.	7.	8.	9.
1. Age	53.5	8.45	-								
2. Experience	22.3	8.48	0.85**	-							
3. IT knowledge/ skills	2.26	0.72	-0.23**	-0.24**	-						
4. Self-efficacy with DMUD	4.46	0.57	0.17*	-0.18*	0.08	-					
5. Experience with DMUD	4.36	0.72	0.07	0.10	0.14	0.54**	-				
6. Experience with Addition	4.35	1.03	-0.04	-0.02	0.00	0.22**	0.62**	-			
7. Interest in DMUD	4.24	0.83	-0.23**	-0.12	0.05	-0.09	0.03	0.13	-		
8. Attitude OT	3.56	0.81	-0.15	-0.13	0.03	-0.21*	-0.14	0.05	0.16	-	
9. Attitude OT for DMUD	3.10	1.00	-0.15	-0.13	0.06	-0.18*	-0.11	0.12	0.21*	.81**	-

Note. *N*= 142. *p < .05. **p <. 01. *OT* = Online Therapy; *DMUD* = Digital media use disorder.

Supplementary Table 5. Overlap of themes and categories in the open-ended questions and answers

Supplementary Table 5a. Therapeutic aspects mentioned as prerequisites, helpful features, and barriers

Therapeutic aspects

Categories	**Prerequisites & Facilitating Factors**	***N***	**Features & Benefits**	***N***	**Barriers**	***N***
Personal contact	• Regular face-to-face contact<br>• Personal contact	25<br>3	Add-on to face-to-face therapy	4	• Lack of therapeutic relationship/ little personal<br>• Lack of non-verbal communication<br>• Add-on to face-to-face therapy	9<br>3<br>1
Structure and control	Specific concepts (e.g. for different disorders, structure of program)	4	• Usage/ Time tracking<br>• Insight into app program/ feedback	1<br>8	Lack of control or feedback about use	6
Parents	Integration of parents/ legal guardians	4				
Age	Age-appropriate programs	3	Age-appropriate programs	1	Age-appropriate programs	1
Commitment and relationship	Strong therapeutic relationship and clear goals	3			• Lack of commitment of patients<br>• Lack of compliance of patients	7<br>4
Interaction	Interactive tools for contact between patient and therapist	2	Interactive functions (e.g. video, chat, group chat)	11		
Emergencies	Handling emergencies (e.g. acute suicidality)	2	Handling emergencies (e.g. acute suicidality)	1	Handling emergencies (e.g. acute suicidality)	3
Digital media					Against more digital media for adolescents (for treatment of DMUD)	7
Content			Psychoeducational content	3		
			Clear structure/modules	3		
			Diaries/visualization of progress	3		
			Relaxation exercises	1		
			Questionnaires	1		
			Skills catalogue	1		

Note. The responses to the questions on prerequisites and solutions to overcome the barriers were clustered for conciseness. Further, the total number of responses per item varied such that total number of responses per theme may vary.

Supplementary Table 5b. Technical aspects mentioned as prerequisites, features, and barriers

Technical aspects

Categories	**Prerequisites & Facilitating Factors**	***N***	**Features & Benefits**	***N***	**Barriers**	***N***
Equipment + problems	Good technical equipment	13	Good technical equipment	2	• Lack of technical equipment<br>• Technical problems	8<br>9
Internet	Stable internet connection	5	Stable internet connection	5	Unstable internet connection	9
Handling	Easily handling, user-friendly design	5	User-friendly, good usability	12		
Training + knowledge	• Training of CAPPs<br>• Sufficient knowledge about technical aspects	6<br>3			Training of CAPPs	1
Access + design	Easy access, e.g. via smartphone	3	Attractive design	8		
IT support	IT support/ hotline	1	IT support/ hotline	1	IT support/ hotline	1

Note. The responses to the questions on prerequisites and solutions to overcome the barriers were clustered for conciseness. Further, the total number of responses per item varied such that the total number of responses per theme may vary.

Supplementary Table 5c. Legal/regulatory aspects mentioned as prerequisites, features, and barriers

Legal/ regulatory aspects

Categories	**Prerequisites & Facilitating Factors**	***N***	**Features & Benefits**	***N***	**Barriers**	***N***
Data protection	Data protection	7	Data protection	4	Data protection	8
Evidence	Evidence-based, validated programs	7	Evidence-based, validated programs	1	Evidence-based, validated programs	1
System	Regulatory aspects	4			Rigid healthcare system	4
Billability	Billability/ compensation	3			Billability/ compensation	3
Platforms	Secure platforms to administer online therapy	2				
(Parental)Consent	Duty of confidentiality/ parental consent	1	Digital informed consent	1	• Digital informed consent<br>• Inclusion of parental/ legal guardian	1<br>2

Note. The responses to the questions on prerequisites and solutions to overcome the barriers were clustered for conciseness. Further, the total number of responses per item varied such that the total number of responses per theme may vary.

Supplementary Table 5d. Organizational aspects mentioned as prerequisites and barriers

Organizational aspects

**Categories**	**Prerequisites, Benefits & Facilitating Factors**	***N***	**Barriers**	***N***
Training	Training opportunities	10	Training of CAPPs	1
Catalogue	Standardized catalogue of available interventions	7		
Facilities	Undisturbed space in facilities	3		
Acceptance & Knowledge	• Acceptance by staff and patients<br>• Reduction of reservations of CAPPs	2<br>4	• Lack of knowledge about online therapy<br>• Low acceptance of CAPPs/ patients<br>• Little willingness to change around CAPPs	2<br>2<br>2
Control	Better controllable in in-patient setting	2		
Lack of time	Time/ effort	2	Lack of time/ effort	6
Testing	Access to test digital interventions	2		
Costs	Costs	1	Financing (e.g. personnel)	2

Note. The responses to the questions on prerequisites, benefits, and solutions to overcome the barriers were clustered for conciseness. Further, the total number of responses per item varied such that the total number of responses per theme may vary.
